# Squalene Loaded Nanoparticles Effectively Protect Hepatic AML12 Cell Lines against Oxidative and Endoplasmic Reticulum Stress in a TXNDC5-Dependent Way

**DOI:** 10.3390/antiox11030581

**Published:** 2022-03-18

**Authors:** Seyed Hesamoddin Bidooki, Teresa Alejo, Javier Sánchez-Marco, Roberto Martínez-Beamonte, Roubi Abuobeid, Juan Carlos Burillo, Roberto Lasheras, Victor Sebastian, María J. Rodríguez-Yoldi, Manuel Arruebo, Jesús Osada

**Affiliations:** 1 Departamento de Bioquímica y Biología Molecular y Celular, Facultad de Veterinaria, Instituto de Investigación Sanitaria de Aragón-Universidad de Zaragoza, E-50013 Zaragoza, Spain; h.bidooki94@gmail.com (S.H.B.); javiersanchezmarc@gmail.com (J.S.-M.); romartin@unizar.es (R.M.-B.); roubi.a.obeid@gmail.com (R.A.); 2 Departamento de Ingeniería Química y Tecnologías del Medio Ambiente, Universidad de Zaragoza, E-50018 Zaragoza, Spain; teresaal@unizar.es (T.A.); victorse@unizar.es (V.S.); arruebom@unizar.es (M.A.); 3 Instituto de Nanociencia y Materiales de Aragón (INMA), CSIC-Universidad de Zaragoza, E-50009 Zaragoza, Spain; 4 Instituto Agroalimentario de Aragón, CITA-Universidad de Zaragoza, E-50013 Zaragoza, Spain; mjrodyol@unizar.es; 5 Centro de Investigación Biomédica en Red de Fisiopatología de la Obesidad y Nutrición (CIBEROBN), Instituto de Salud Carlos III, E-28029 Madrid, Spain; 6 Laboratorio Agroambiental, Servicio de Seguridad Agroalimentaria de la Dirección General de Alimentación y Fomento Agroalimentario, Gobierno de Aragón, E-50059 Zaragoza, Spain; jcburillo@aragon.es (J.C.B.); rjlasheras@aragon.es (R.L.); 7 Centro de Investigación Biomédica en Red de Bioingeniería, Biomateriales y Nanomedicina (CIBER-BBN), Instituto de Salud Carlos III, E-28029 Madrid, Spain; 8 Departamento de Farmacología, Fisiología, Medicina Legal y Forense, Facultad de Veterinaria, Instituto de Investigación Sanitaria de Aragón-Universidad de Zaragoza, E-50013 Zaragoza, Spain

**Keywords:** olive oil, liver, squalene, PLGA, oxidative stress, endoplasmic reticulum stress, TXNDC5, *Gpx4*, *Ern1*, *Eif2ak3*

## Abstract

Virgin olive oil, the main source of fat in the Mediterranean diet, contains a substantial amount of squalene which possesses natural antioxidant properties. Due to its highly hydrophobic nature, its bioavailability is reduced. In order to increase its delivery and potentiate its actions, squalene has been loaded into PLGA nanoparticles (NPs). The characterization of the resulting nanoparticles was assessed by electron microscopy, dynamic light scattering, zeta potential and high-performance liquid chromatography. Reactive oxygen species (ROS) generation and cell viability assays were carried out in AML12 (alpha mouse liver cell line) and a TXNDC5-deficient AML12 cell line (KO), which was generated by CRISPR/cas9 technology. According to the results, squalene was successfully encapsulated in PLGA NPs, and had rapid and efficient cellular uptake at 30 µM squalene concentration. Squalene reduced ROS in AML12, whereas ROS levels increased in KO cells and improved cell viability in both when subjected to oxidative stress by significant induction of *Gpx4*. Squalene enhanced cell viability in ER-induced stress by decreasing *Ern1* or *Eif2ak3* expressions. In conclusion, TXNDC5 shows a crucial role in regulating ER-induced stress through different signaling pathways, and squalene protects mouse hepatocytes from oxidative and endoplasmic reticulum stresses by several molecular mechanisms depending on TXNDC5.

## 1. Introduction

The Mediterranean diet, also known as the Med diet, has been associated with a variety of health benefits on cardiovascular diseases, including a reduction in the frequency of cardiovascular events as well as their risk factors, including obesity and metabolic disorders such as diabetes, hypertension and dyslipidemia [1]. This diet pattern consists of varying plant-based food sources such as fruits, vegetables, olive oil and nuts [2]. Virgin olive oil, the main source of fat in this diet, has been linked to a reduced risk of general and cause-specific mortality [3]. It is composed of an oily matrix of triglycerides containing monounsaturated fatty acids and a minor fraction dubbed unsaponifiable [4,5]. The biological effects of the latter compounds have recently been the focus of attention [6].

Squalene accounts for almost 90% of the hydrocarbons present in the unsaponifiable fraction of virgin olive oil [7]. Squalene is a terpenoid-like natural lipid with an isoprenoid structure that is used as an intermediary in the biosynthesis of phytosterols and terpenes in plants and cholesterol in animals [8]. Squalene has a number of demonstrated therapeutic features, including being a natural antioxidant, lowering blood cholesterol levels and having tumor-protective properties [9]. For instance, squalene inhibited aberrant hyperproliferation in a non-tumorigenic mammary epithelial cell line in vitro, according to Katdare et al. [10]. Squalene suppressed cell proliferation in an invasive MDA-MB-231 breast cancer cell line by triggering apoptosis and DNA damage [11]. Murakoshi et al. found that topically applied squalene significantly reduced mice skin tumors [12].

Several compounds encapsulated in nanoparticles for anti-cancer therapy and other disorders have been extensively studied with the goal of protecting sensitive chemicals from degradation, increasing their solubility and therefore favoring their bioavailability [13,14]. Nanoparticles have also been used for targeting and crossing biological barriers, lessening irritation or facilitating the bioavailability of different drugs while minimizing side effects [15,16]. As a result, a wide range of naturally derived and biodegradable particles, such as chitosan, poly (lactic-co-glycolic acid) (PLGA) and protein-based particles have been developed [17]. PLGA is one of the most effectively used biodegradable polymers due to the fact that its hydrolysis results in endogenous metabolite monomers, lactic acid and glycolic acid, which can easily be degraded in the body by the Krebs cycle. Therefore, because the body can adequately metabolize these two monomers, the use of PLGA for drug delivery or tissue engineering applications has a low risk of systemic toxicity [18]. Many routes have been reported to be involved in PLGA nanoparticle cellular uptake and penetration into the cytoplasm [19,20].

Protein synthesis, processing, and folding; intracellular transport and calcium signaling; drug detoxification; and lipid metabolism are all performed in the endoplasmic reticulum (ER), which is a multifunctional organelle [21]. ER homeostasis alterations are caused by high levels of free fatty acids, calcium depletion or insulin resistance and lead to an accumulation of misfolded proteins, which triggers the unfolded protein response (UPR) [22,23,24]. Grp78 releases Ern1, Eif2ak3 and Atf6 as a result of the presence of unfolded proteins in the ER [25,26]. Squalene reduces hepatic fat content and induces the expression of proteins involved in the lipidic metabolism [27]. Our hypothesis is that the restoring of those critical proteins by administering bioavailable squalene may result in reduced ER stress.

The imbalance between the excessive generation of cellular reactive oxygen species (ROS) [26] and the reduced ability of live organisms to counteract ROS through antioxidant system response is commonly referred to as oxidative stress [28,29,30]. Research from animal models suggests that oxidative stress plays a role in steatohepatitis [31]. Squalene’s function has been studied in a variety of cell lines and appears to be linked to quenching oxidative stress. In mouse peritoneal macrophages and human promyelocytic leukemia cell lines (HL-60), respectively, squalene reduced the intracellular ROS content caused by lipopolysaccharide incubation and also suppressed hydrogen peroxide-induced protein carbonylation [32,33]. Squalene reduced intracellular ROS levels, inhibited H_2_O_2_-induced oxidative injury and protected human mammary epithelial cells (MCF10A) against oxidative DNA damage [34]. The liver is the most commonly affected organ by oxidative stress, owing to its constant exposure to oxidative stimuli and its high mitochondrial activity [30,35]; dietary squalene administration could reduce oxidative stress in several mice models as well [36].

Thioredoxin domain-containing 5 (TXNDC5) protects hepatic cells from stress-induced apoptosis [37,38]. TXNDC5 is situated in the ER and, as a member of the protein disulfide isomerase (PDI) family, is implicated in protein modification and folding [39]. During hypoxic situations, TXNDC5 is abundantly expressed in the liver and endothelial cells and performs vital roles in anti-oxidative harm, anti-anoxia-induced apoptosis and cellular proliferation [40,41]. Hence, this present study describes the protection function of PLGA-based squalene nanoparticles on oxidative and ER stress in mouse hepatocytes. To address these issues and acquire a better understanding of the mechanisms involved in the putative role of squalene, the function of TXNDC5 and the main ER molecular mechanisms in stress circumstances were explored.

## 2. Materials and Methods

### 2.1. Preparation of PLGA-Based Squalene-Loaded Nanoparticles

Squalene–PLGA polymeric nanoparticles were synthesized by the single-emulsion solvent evaporation technique [42,43] using Resomer^®^ RG 503H poly(D,L-lactide-co-glycolide) (PLGA-COOH, Mw 24–38 kDa) (Sigma-Aldrich; Merck Millipore, Darmstadt, Germany), Pluronic F68 (Panreac Química S.L.U; Barcelona, Spain) and ethyl acetate 99.6% ACS (Sigma-Aldrich, Merck Millipore, Darmstadt, Germany) in the presence of 100, 75, 50, 25 µL of squalene (2.05 M, ≥98%, liquid) (Sigma-Aldrich, Merck Millipore, Darmstadt, Germany). Briefly, PLGA (50 mg) and Pluronic (150 mg) were dissolved in ethyl acetate (5 mL). Different concentrations of squalene were added to the solution together with 10 mL of Milli-Q water and sonicated (Branson Digital Sonifier 450, Danbury, CT, USA) in an ice bath for 25 s and at 40% amplitude using a probe of 0.13 inches in diameter. Then, the organic solvent was evaporated under sterile conditions for 3 h with stirring at 600 rpm. Finally, the nanoparticles were collected by centrifugation (Thermo Fisher Scientific, Waltham, MA, USA) at 12,350× *g* and then at 15,000× *g* for 15 min at 10 °C and dispersed in fresh PBS for the subsequent cellular experiments.

### 2.2. Physicochemical Characterization of the Nanoparticles

A scanning electron microscope (SEM, FEG INSPECT-F50, Eindhoven, Netherlands) was used to determine the morphology of the resulting nanoparticles. For sample preparation, a drop of the NP dispersion (10 µL, 1 mg/mL) was placed on a glass slide, fixed with carbon tape to a holder, air-dried overnight and sputtered with a very thin, fine-grained Palladium coating to facilitate electron conduction (Leica EM ACE200, Wetzlar, Germany). In order to determine the resulting particle size, particles were also analyzed using transmission electron microscopy (TEM) (Tecnai T20, FEI Company, Hillsboro, OR, USA, operating at 200 kV). This microscope is equipped with a thermionic gun (LaB6) and a SuperTwin^®^ objective lens that allows a 0.24 nm spatial resolution. The CCD camera selected to take the TEM Images was a Veleta CCD 2k × 2k, for fast acquisition and a wide field of view. Squalene–PLGA NPs were negatively stained using phosphotungstic acid dissolved in Milli-Q water (30 mg/mL). TEM samples were prepared on Forward Cu-200 mesh TEM grids by depositing 100 µL (1 mg/mL) of NP dispersion onto the grid, then squalene–PLGA NPs were stained with a phosphotungstic acid solution, washed with water to remove salts in excess and finally dried overnight. For each sample, four different areas of the grid were examined to obtain representative results, obtaining at least 15 images per sample. At least 150 nanoparticles were measured from TEM images using ImageJ software version 3.5 to plot the particle size histogram and determine mean diameter and standard deviation. A Brookhaven 90 Plus (Holtsville, NY, USA) (90° scattering angle, 25 °C) was used to assess the hydrodynamic particle size and zeta potential of the nanoparticles in water at neutral pH. When suspending 20 µL of NPs in 3 mL of distilled water for size measurements by dynamic light scattering (DLS), a good attenuator value (7–9) was procured. The average of five 180 s measurements yielded the mean hydrodynamic diameter for each preparation. PLGA-squalene NPs (70 µL) were dispersed in 2 mL of 1 mM KCl before filling the measurement cell for zeta potential measurements at neutral pH and 25 °C. The average of five independent measurements in automated mode, followed by the application of the Smoluchowski equation, yielded the mean zeta potential for each preparation. The findings were standardized using blank PLGA nanoparticles without squalene as a reference.

### 2.3. PLGA Encapsulation Efficiency Experiment

In a 5% dextrose solution, PLGA NPs with different initial squalene contents (100, 75, 50, 25 µL of 2.05 M of squalene) were formulated and then washed twice with water using an ultrafiltration device (Amicon, molecular weight cut-off, 100,000 Da). Squalene was extracted from the NPs using ethanol and quantified by high-performance liquid chromatography (HPLC). The HPLC Waters Alliance 1695 (Waters, Milford, MA, USA) was used, which was retrofitted with a Waters DAD 2996 photodiode array detector, a Hewlett-Packard computer running Waters Empower 3 software and a Waters autosampler with a 50 µL loop. Synchronous spectra detection wavelengths ranging from 200 to 600 nm were recorded for all peaks. A non-gradient mobile phase of acetonitrile and methanol (50:50, *v*/*v*) was adopted at a constant flow rate of 1 mL/min on a Waters XSelect LC-18 column (2.1 mm by 150 mm, 3.5 µm). The squalene peak was calculated quantitatively by comparing it to a standard curve at a wavelength of 216 nm. The absorbance of the organic solvents in the selected wavelength of 216 nm was subtracted by the autozero.

### 2.4. Squalene Extraction

Following cell harvest and squalene being loaded into PLGA NPs, squalene was extracted and analyzed by gas chromatography and mass spectrometry (GC/MS), as previously described [44].

### 2.5. AML12 Cell Culture

The mouse hepatocyte cell line (AML12) was obtained from the ATCC collection (Manassas, VA, USA) and cultured in a 6-well plate (in duplicate) at 37 °C in a humidified atmosphere of 5% CO_2_ in Dulbecco’s modified Eagle’s minimum essential medium (DMEM; Thermo Fisher Scientific, Waltham, MA, USA): F-12-Ham’s medium (GE Healthcare Life Science, South Logan, UT, USA) at a 1:1 ratio supplemented with 10% fetal bovine serum (Thermo Fisher Scientific, Waltham, MA, USA), 1:500 insulin-transferrin-selenium (Corning, Bedford, MA, USA), 40 ng/mL dexamethasone (Sigma-Aldrich; Merck Millipore, Darmstadt, Germany), 1% nonessential amino acids (Thermo Fisher Scientific, Waltham, MA, USA), 1% amphotericin B (1000 mg/mL; Thermo Fisher Scientific, Waltham, MA, USA), 1% penicillin (1000 U/mL; Thermo Fisher Scientific) and 1% streptomycin (1000 mg/mL; Thermo Fisher Scientific, Waltham, MA, USA). This medium was removed after the AML12 cells reached 90–100% confluence, and the cells were washed once with PBS before being given the medium free of fetal bovine serum and amphotericin B. For RNA isolation and cDNA synthesis, performed as described below, cells were treated with 12.5 nM of thapsigargin (Sigma-Aldrich, Merck Millipore, Darmstadt, Germany) or 25 mM of H_2_O_2_ (Sigma-Aldrich, Merck Millipore, Darmstadt, Germany) for 24 h and 30 min, respectively, after 72 h of exposure to 30 µM of squalene loaded PLGA nanoparticles.

### 2.6. Characterization of Cell Morphology in Presence of PLGA-Squalene Nanoparticles

AML12 cells (2000 cells per well) were cultured in a 24-well plate (in duplicate). Cells were incubated for 72 h in the presence of 150, 60, 30 and 15 µM squalene loaded in PLGA nanoparticles and non-loaded PLGA nanoparticles as control. After washing 2 times with PBS, cells were mixed with 4% formaldehyde (Panreac Química S.L.U., Barcelona, Catalonia, Spain) and dissolved in PBS for 30 min at room temperature. Cells were washed with 60% isopropanol (Panreac Química S.L.U., Barcelona, Catalonia, Spain) and PBS, respectively, afterward, stained with 1% Nile Red (Thermo Fisher Scientific, Waltham, MA, USA) and dissolved in PBS for 15 min in the dark. After PBS wash, an epifluorescence microscope (Floid Cell Imaging System; Thermo Fisher Scientific, Waltham, MA, USA) with excitation and emission wavelengths of 552/636 nm was utilized for detecting the influence of several PLGA NPs concentrations on the AMl12 cell line.

### 2.7. Generation of a Stable TXNDC5 Knockout AML12 Cell Line

The AML12 cell line was grown as erstwhile explained to create stable clones without TXNDC5. The culture medium was withdrawn after one week of development, and the cells were washed twice with PBS before being transfected with TXNDC5/ERp46 HDR and TXNDC5 CRISPR/Cas9 KO plasmids (Santa Cruz Biotechnology, Dallas, TX, USA) using lipofectamine 2000 (Thermo Fisher Scientific, Waltham, MA, USA). TXNDC5 CRISPR/Cas9 KO plasmid possesses gRNA sequence; 5′-TTATCAAGTTCTTCGCTCCG-3′ to generate a double-stranded break (DSB) specifically in the fifth exon of *Txndc5*. To provide the selection of constant knockout (KO) AML12 cells, the TXNDC5/ERp46 HDR recombined the *Txndc5* gene containing a puromycin resistance gene. Puromycin-resistant AML12 KO cells were selected after several rounds of puromycin incubations. TXNDC5 absence was confirmed by Western blot (Supplementary Figure S1).

### 2.8. RNA Extraction

Total cellular RNA was extracted according to the manufacturer’s instructions by using a Quick-RNA^TM^ MiniPrep kit (Zymo Research, CA, USA). RNA was quantified based on the absorbance ratio at 260/280 nm wavelength using a Nanodrop 2000c Spectrophotometer (Thermo Fisher Scientific, Waltham, MA, USA). The integrity of the 28S and 18S ribosomal RNAs was confirmed by electrophoresis on a 1% agarose gel followed by ethidium bromide staining, and the 28S/18S ratio was larger than 2.

### 2.9. Quantitative Real-Time PCR (RT-qPCR)

To achieve equivalent efficiencies, the reverse transcriptase quantitative PCR tests of these transcripts were optimized in terms of primer and input cDNA concentrations. The PrimeScript RT reagent kit (TaKaRa Biotechnology, Kusatsu, Shiga, Japan) was used to reverse transcribe 500 ng of extracted total RNA into the supplementary deoxyribonucleic acid in the presence of random and oligo (dT) primers, following the manufacturer’s instructions. Primer Express (Applied Biosystems, Foster City, CA, USA) was used to design the primers for each gene, as mentioned in Supplementary Table S1, which were then validated for gene specificity and amplification of cDNA rather than genomic DNA using BLAST analysis (NCBI); eventually, the primers were selected based on the primer efficiency. On a Step One Plus Real-Time PCR System (Applied Biosystem, Foster City, CA, USA), quantitative real-time PCR was performed according to the manufacturer’s guidelines (SYBR Green PCR Master Mix, Applied Biosystems, Foster City, CA, USA). Each gene’s transcript expression level was estimated using the comparative 2^−ΔΔCT^ method, normalized to the endogenous control genes *Ppib* and *Tbp* and expressed as a relative ratio to the mean values of control samples.

### 2.10. Western Blot

The Bradford reagent (Bio-Rad, Hercules, CA, USA) was used to assess the total protein concentration in AML12 WT and KO cells after they were lysed. Then, 10 µg of proteins were separated on a 10% sodium dodecyl sulfate-polyacrylamide gel electrophoresis and transferred to polyvinylidene difluoride filter membranes (Bio-Rad, Hercules, CA, USA). The membrane was blocked for 1 h at room temperature using a PBS buffer containing 5% BSA. After blocking, the membrane was incubated at 4 °C overnight with a primary rabbit polyclonal antibody against mouse TXNDC5 (1:1000, Proteintech, Manchester, UK) and mouse monoclonal anti-β-ACTIN (1:1000, Sigma, St Louis, MO, USA). The membrane was washed three times with a PBS buffer containing 0.1% Tween 20 and incubated for 1 h at room temperature with conjugated goat anti-rabbit IgG (H&L) DyLight 800 secondary antibody (1: 60,000, Thermo-scientific, Waltham, MA, USA) and goat anti-mouse IgG (H&L) DyLight 680 secondary antibody (1: 30,000, Thermo-scientific, Waltham, MA, USA). Blot was visualized by Odyssey^®^Clx (LI-COR, Bad Homburg, Germany).

### 2.11. Cell Viability Assay

Cell viability was determined using 3-(4 5-dimethylthiazol-2-yl)-2 5-diphenyltetrazolium bromide assay (MTT; Sigma-Aldrich, Merck Millipore, Darmstadt, Germany). Cells were seeded on a 96-well plate at 5000 cells/well and exposed for 24 h to 18 nM of thapsigargin (Sigma-Aldrich, Merck Millipore, Darmstadt, Germany) dissolved in 0.1% DMSO for ER stress and 30 min of 20, 25 and 30 mM of H_2_O_2_ (Sigma-Aldrich, Merck Millipore, Darmstadt, Germany) for oxidative stress in presence of 30 µM of nanoencapsulated squalene for 72 h, thereupon 1 mg/mL of MTT was added to the culture medium. Following 3 h incubation, cell growth medium was replaced by DMSO and absorbance measurements were assessed with a 96-well plate reader at 570 nm.

### 2.12. Reactive Oxygen Species Assay

AML12 cells (5000 cells per well) were seeded in a 96-well plate and cultured for 72 h at 37 °C. The cells were treated for 72 h with PLGA-squalene NPs or control NPs diluted in the medium free of fetal bovine serum and amphotericin B at a concentration of 30 µM; afterward, 10 µL of 2.0 mg/mL 2,7-dichlorofluorescein diacetate (DCFH-DA; Sigma-Aldrich, Merck Millipore, Darmstadt, Germany) dissolved in fresh PBS were added to the cells. After 3 h, the medium was removed, and cells were incubated with a medium containing hydrogen peroxide (H_2_O_2_; final concentration, 25 mM; Sigma-Aldrich, Merck Millipore, Darmstadt, Germany). After 3 h, the presence of ROS was assessed by measuring the conversion of DCFH-DA into fluorescent dichlorofluorescein (DCF) at excitation and emission wavelengths of 485 and 520 nm in a microplate reader (FLUOstar^®^, Omega, BMG Labtech, Ortenberg, Germany), respectively.

### 2.13. Statistical Analysis

Statistical analyses were carried out using the GraphPad Prism 8 for Windows (GraphPad, S. Diego, CA, USA). Statistical significance was defined as a *p*-value of less than 0.05. The Mann–Whitney U test was used to conduct the statistical analysis. The Shapiro–Wilk test was used to determine the normal distribution of data and Bartlett’s or Levene’s tests were used to determine the homology of variance among groups. A 2-tailed Student’s t-test and two-way ANOVA with Dunnett’s multiple comparisons test were used to investigate parameters that matched both criteria. The means and standard deviations of the results are shown.

## 3. Results

### 3.1. Synthesis and Physicochemical Characterization of Squalene Loaded PLGA Nanoparticles

**Figure 1:**
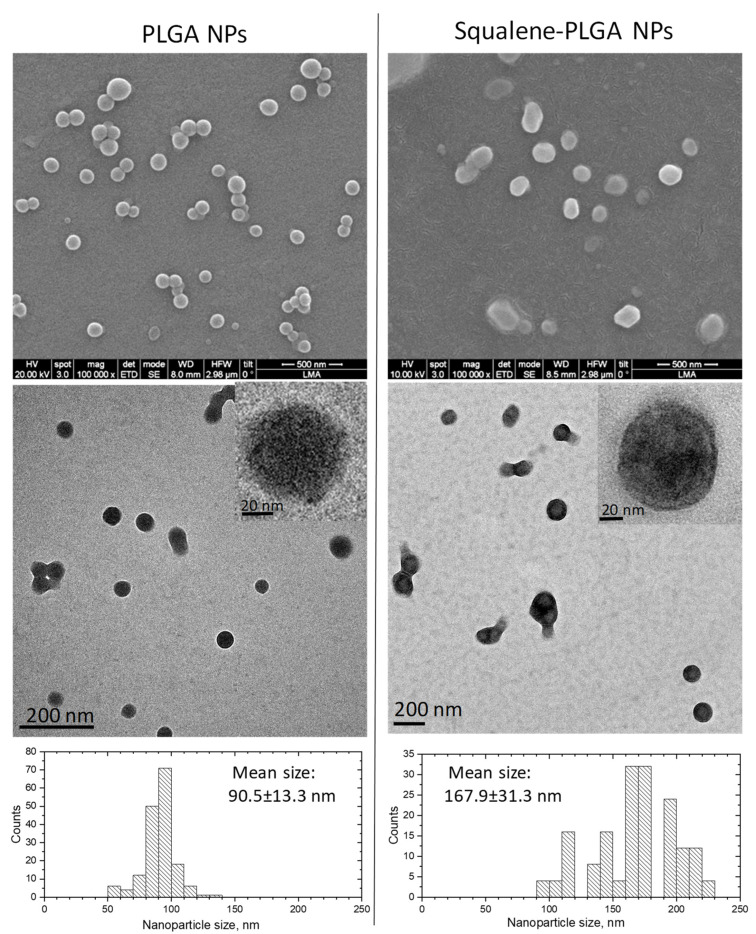
Electron microscopy analysis, SEM, TEM and particle size histograms of PLGA nanoparticles with and without squalene.

To examine the synthesis and physicochemical characterization of the nanoparticles prepared using the single emulsion approach, the squalene encapsulation ability of PLGA was characterized by HPLC (Table 1). The maximum squalene loading in PLGA-based nanoparticles was found when using 50 µL initial squalene (2.05 M, ≥98%, liquid) volume that reached 8122 ± 735 µM of squalene loading. This represented 77.8 ± 5.1% encapsulation efficiency, and the highest ratio of squalene referred to PLGA of the ones tested. The encapsulation efficiency reported in the literature for squalene loaded nanoparticles was variable depending on the nanoparticle type and the synthetic method ranging from 26 to 82% [45,46,47]. These nanoparticles were selected to carry out the experimental work. Nanoparticles were characterized by scanning electron microscopy (SEM) to determine their morphology and size (Figure 1). PLGA NPs devoid of squalene were spherical in shape, in contrast to NPs loaded with squalene that showed a larger size an oval shape (Figure 1). Transmission electron microscopy (TEM) analysis confirmed a general trend toward size increase when squalene was loaded in PLGA (Figure 1). Particle size histograms determined from TEM images revealed that the mean size of PLGA nanoparticles increased from 90.5 ± 13.3 to 167.9 ± 31.3 when squalene was encapsulated (Figure 1). Then, it seems that squalene affects both the micelle shape and size during the emulsification process. This observation is in agreement with previous results where the presence of lipids such as cholesterol can increase the PLGA size [48]. The hydrodynamic diameter of the nanoparticles in water at neutral pH, estimated by dynamic light scattering (DLS), was 259 ± 107 nm for squalene loaded PLGA NPs and their dispersion was higher than that of empty NPs (Table 2). The analysis on the particle size between TEM and DLS demonstrated the same trend, confirming the size enlargement when squalene is encapsulated. The nanoparticle mean diameters from TEM image-based measurements were smaller than the ones obtained from hydrodynamic size measurement. However, these differences can be explained by the technique differences—in TEM, the nanoparticles are dried, and the size should be smaller after shrinking [49,50,51,52]. The presence of squalene also had a clear impact on the zeta potential values. In this sense, the negative values (−46.8 ± 0.6 mV) of PLGA nanoparticles without squalene significantly decreased in PLGA-based squalene nanoparticles (−36.2 ± 0.3 mV) at neutral pH. The negative charge of PLGA NPs without squalene could be attributed to the presence of ionized carboxyl groups in the acid terminated PLGA (PLGA-COOH), whereas the less negative charge of PLGA-based squalene nanoparticles might be due to the presence of squalene on the surface of the nanoparticles, as described previously for other nanoparticles [53].

### 3.2. Influence of Several PLGA NPs Concentrations on the Morphology of AMl12 Cell Line

**Figure 2:**
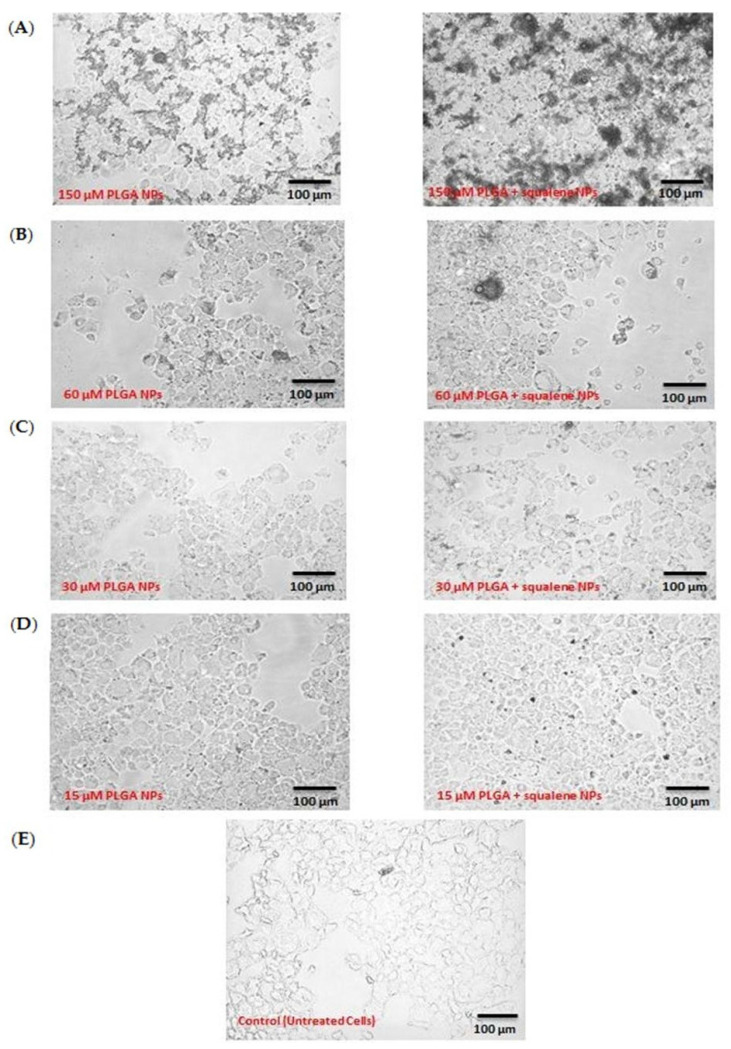
Detection of the effect of different PLGA nanoparticles concentrations (with and without squalene) on the AML12 cell line. (**A**) 150 µM, (**B**) 60 µM, (**C**) 30 µM, (**D**) 15 µM and (**E**) untreated cells.

After squalene extraction and GC/MS analysis, the squalene concentration loaded into PLGA nanoparticles was determined to be 3.05 mM. The influence of various concentrations of PLGA nanoparticles was qualitatively examined by microscopy after 72 h of incubation with AML12 cells. Hence, four different concentrations were investigated. Figure 2 shows the AML12 cell line after exposure to 150, 60, 30 and 15 µM doses of squalene loaded into PLGA nanoparticles and to the PLGA nanoparticles without squalene. The 150 µM PLGA NPs caused the cell to completely shrink (Figure 2A), but the shrinkage was mitigated by lowering the nanoparticle dosage (Figure 2B–D). Due to the observed morphological impact of the highest doses, the 30 µM of squalene loaded in the PLGA nanoparticles was selected to carry out the subsequent studies on AML12 cells.

### 3.3. AML12 Cellular Uptake of Squalene

**Figure 3:**
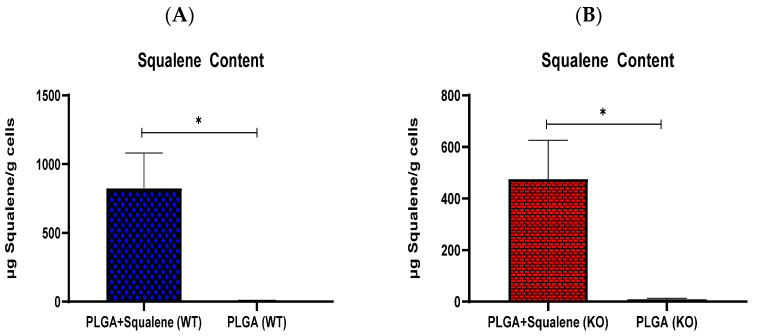
In vitro cellular uptake of squalene. Hepatic AML12 cells were incubated with 30 μM of PLGA-based squalene NPs and PLGA NPs for 72 h. (**A**) normal mouse AML12 cells (wild-type (WT)), (**B**) TXNDC5-deficient AML12 cells (knockout (KO)). Statistical analyses were done according to Mann–Whitney’s U-test; * *p* < 0.05.

To study the capability of PLGA-based squalene NPs to be uptaken by the AML12 hepatocyte cell line, these cells were incubated in the presence of 30 µM of PLGA-based squalene NPs and empty PLGA NPs for 72 h, and cellular squalene content was measured (Figure 3). As shown, the cellular uptake of squalene increased significantly when the NPs were loaded with squalene, indicating that this is an efficient vehicle to deliver this compound in this cell line.

### 3.4. Squalene Based PLGA NPs Could Effectively Protect against Oxidative Stress

**Figure 4:**
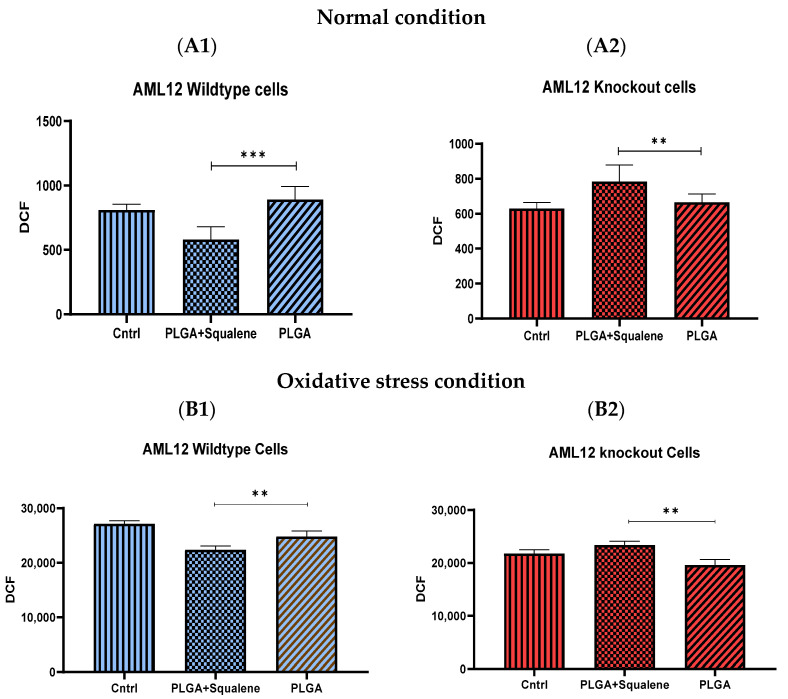
Assessment of ROS production in normal mouse AML12 cells cell line (AML12 wild-type (WT)) and TXNDC5-deficient AML12 cells (AML12 knockout (KO)). (**A**,**B**) After treatment of cells with 30 µM of squalene NPs for 72 h, (**A**) ROS was measured in normal conditions, (**B**) oxidative stress circumstance by 25 mM of H_2_O_2_ for 3 h. (**A1**) potent reduction of ROS in squalene group in WT cells, and (**A2**) significant enhancement in KO cells were observed. (**B1**) Considerable decrement of ROS in squalene group in WT cells and (**B2**) remarkable increase in KO cells are indicated. Statistical analyses were done according to Mann–Whitney’s U-test; ** *p*< 0.01, *** *p*< 0.001.

Normal mouse hepatocyte AML12 cells (wild-type (WT)) and TXNDC5-deficient AML12 cells (knockout (KO)) were incubated with 30 µM of squalene loaded NPs for 72 h to assess the induction of intracellular ROS by PLGA-based squalene nanoparticles, and the amount of intracellular ROS was calculated according to the dichlorodihydrofluorescein (DCF) production. As shown in Figure 4(A1), WT cells treated with squalene NPs exhibited a significant decrease in ROS production compared with the PLGA group. However, squalene NPs increased the formation of ROS in KO samples (Figure 4(A2)). The comparison of the control group and the PLGA illustrates that PLGA nanoparticles did not induce ROS in both cell lines. To peruse whether 30 µM of squalene loaded NPs could effectually represent protection against oxidative stress, we initially developed an in vitro model of oxidative insult. When WT and KO cells were incubated with 25 mM H_2_O_2_ for 3 h, a significant reduction in intracellular reactive oxygen species was detected in AML12 WT cells treated with squalene NPs (Figure 4(B1)), whereas in the KO cells, the increment was observed (Figure 4(B2)). These results suggest that there was not any squalene protection in cells lacking TXNDC5 in exposure to the oxidative agent.

**Figure 5:**
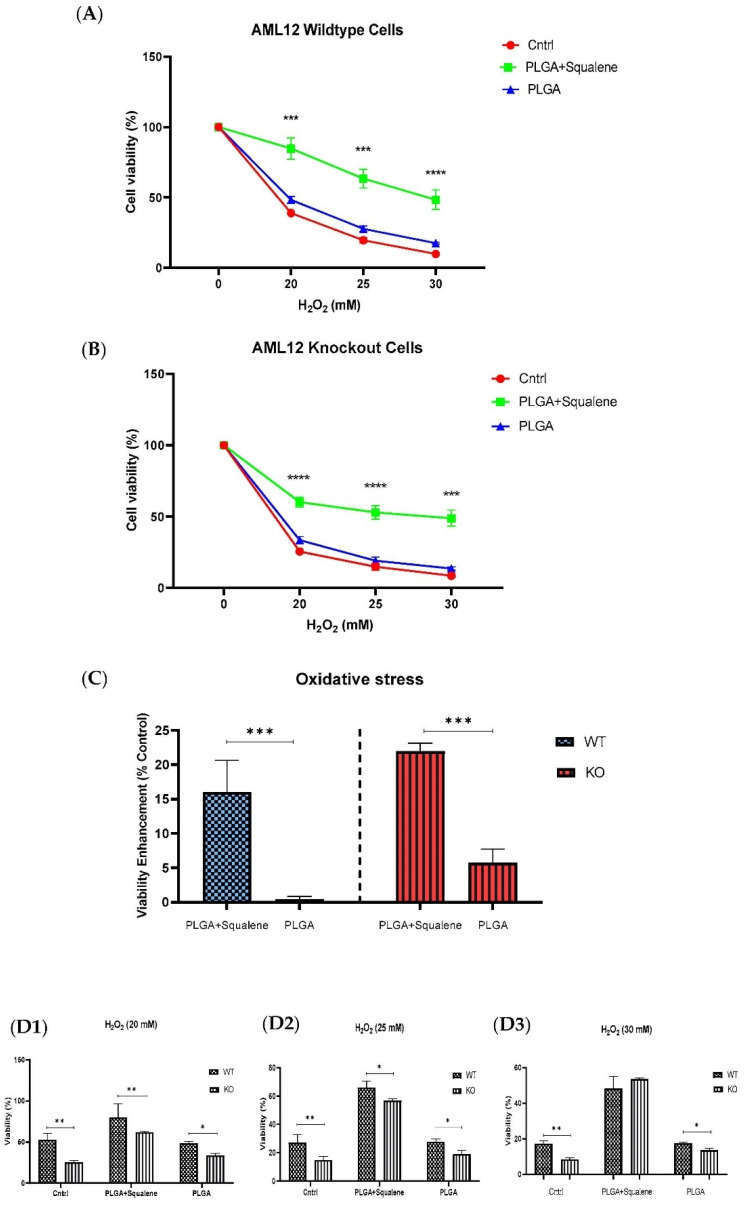
Squalene loaded PLGA NPs increased viability against oxidative stress. MTT was applied to evaluate the cell viability. (**A**) Normal mouse AML12 cells (AML12 wild-type (WT)) were exposed to 30 µM PLGA based squalene NPs for 72 h had a significant increase in viability in presence of 20, 25 and 30 mM of H_2_O_2_, and (**B**) TXNDC5-deficient mouse hepatocyte cells (AML12 knockout (KO)) displayed a similar increment in viability. (**C**) Statistically, a significant difference of 16% and 21% was observed on average in viability enhancement of WT and KO cell lines, respectively, related to respective control in 20, 25 and 30 mM of H_2_O_2_. (**D**) TXNDC5 deletion can drastically lower the viability of the mouse hepatocyte in the absence of squalene at all concentrations tested; however, when the cells were treated with squalene, the viability of both cell lines increased. Although (**D3**) there were no significant differences between WT and KO cells at 30 mM H_2_O_2_, (**D1**,**D2**) a statistical difference was seen in samples treated with squalene loaded in PLGA nanoparticles in presence of 20 and 25 mM H_2_O_2_. Statistical analysis was carried out according to two-way ANOVA and Mann–Whitney’s U-test for pairwise comparisons; * *p* < 0.05, ** *p* < 0.01, *** *p* < 0.001, **** *p* < 0.0001.

The cell viability of WT and KO cells is shown in Figure 5. After 72 h exposure to 30 µM concentrations of both mixtures of PLGA and of the squalene loaded into the PLGA nanoparticles, the cells were treated with three different concentrations of H_2_O_2_ for 30 min. The PLGA NPs did not produce notable alteration on the cell viability, according to the MTT assay, when compared to the control group. Squalene loaded PLGA NPs could significantly increase the viability of both cell lines (Figure 5A,B). Viability enhancement was calculated in the different H_2_O_2_ concentrations that were tested, and the results showed that the squalene delivered from PLGA nanoparticles incremented, on average, the viability in the wild-type mouse hepatocyte cells and knockout by 16 and 21%, respectively (Figure 5C).

### 3.5. Effect of Squalene Protection on Oxidative Stress Response In Vitro by Gpx4 Induction

**Figure 6:**
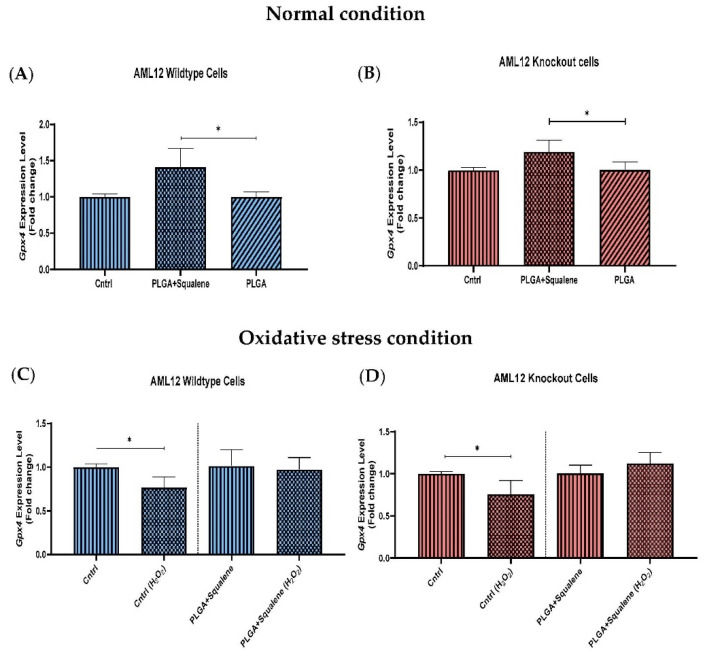
Effect of 30 µM PLGA based squalene nanoparticles on the *Gpx4* gene expression levels of oxidative-challenged (**A**,**C**) in wild-type mouse hepatic cells, and (**B**,**D**) knockout mouse hepatic cells. (**A**,**B**) Significant *Gpx4* induction after 72 h treatment with squalene encapsulated by PLGA in WT and KO cell line, respectively, (**C**,**D**) H_2_O_2_ can significantly downregulate *Gpx4* mRNA level after 30 min exposure to the 25 mM in the control group; whereas squalene encapsulated by PLGA prevented this reduction in both cell lines. The Mann–Whitney U-test was used in the statistical analysis; * *p* < 0.05.

After treatment for 72 h with squalene-loaded PLGA based NPs (30 µM), we examined the levels of glutathione peroxidase 4 in both cell lines. Specifically, we discovered that *Gpx4*, which is involved in the antioxidant defense to neutralize oxidative stress, significantly increased its mRNA level in the squalene-treated samples (Figure 6A,B). Compared with the control group (Figure 6C,D), mRNA levels of *Gpx4* were significantly downregulated by the oxidative challenge (25 mM H_2_O_2_), irrespective of squalene supplementation. However, in squalene-treated samples, there was not any considerable reduction in mRNA levels of *Gpx4* in the presence of hydrogen peroxide.

### 3.6. Squalene Enhanced the Cell Viability of Mouse Hepatocytes in Harsh ER Stress

**Figure 7:**
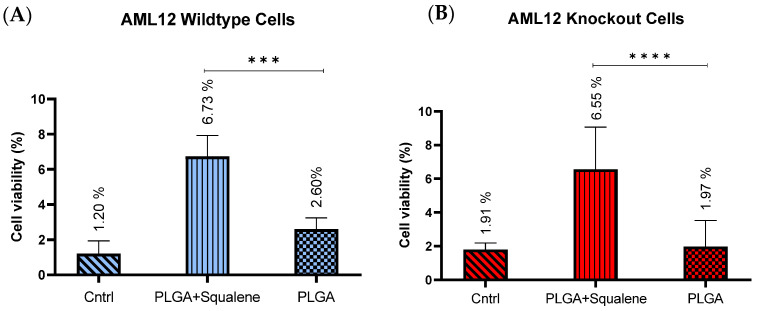
The cell viability evaluation of mouse hepatocytes upon ER stress. After 72 h, treated with 30 µM of squalene-loaded PLGA nanoparticles, ER stress was induced by 18 nM of thapsigargin for 24 h. (**A**,**B**) PLGA NPs did not produce a substantial difference in cell viability when compared to the untreated cells. PLGA based squalene nanoparticles at the 18 nM of thapsigargin (**A**) produced an enhancement of approximately 6% on the cell viability in WT cells (cell viability is 2.60% in control); likewise, (**B**) similar result was observed in KO cells. Statistical analyses were conducted according to two-way ANOVA; *** *p* < 0.001, **** *p* < 0.0001.

ER stress is an adaptive stress response program that is induced by thapsigargin. In AMl12 cells, MTT revealed an IC_50_ of 12.5 nM for thapsigargin. The cell viability under harsh ER stress conditions was evaluated after 72 h of treatment with 30 µM squalene loaded in PLGA and 24 h exposure to the 18 nM of thapsigargin. The PLGA nanoparticles without the squalene revealed no changes (Figure 7). After exposure to ER stress, both cell lines (i.e., AML12 wild-type and knockout) exhibited a fairly remarkable increase in the cell viability when using squalene-containing nanoparticles, i.e., at 18 nM cell viability reached values of 6.73% for WT (Figure 7A) and 6.55% for KO in average (Figure 7B), compared to the unexposed control.

### 3.7. Squalene Protects Thapsigargin-Induced ER Stress by Ern1 and Eif2ak3 Pathways

**Figure 8:**
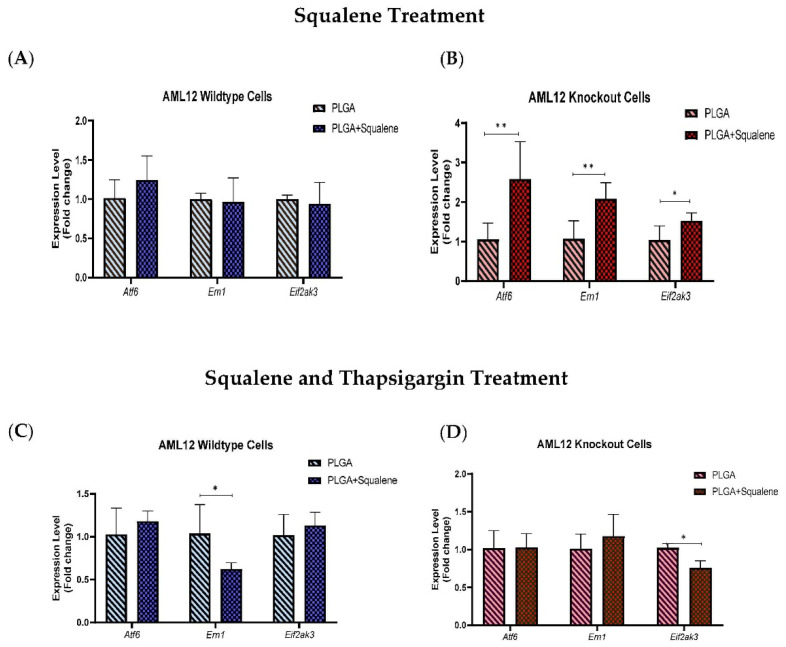
The mRNA expressions of *Atf6*, *Ern1* and *Eif2ak3* in presence of squalene-PLGA nanoparticles under the ER stress challenge. After 72 h of treatment with 30 µM PLGA based squalene NPs, (**A**) the results of three genes (*Atf6*, *Ern1* and *Eif2ak3*) represented a non-significant difference in WT cells. (**B**) Squalene with PLGA NPs induced a striking discrepancy in mRNA levels of *Atf6*, *Ern1* and *Eif2ak3* in TXNDC5 knockout cells. In order to evaluate the protective activity of squalene-PLGA NPs, when the cells were treated with 12.5 nM thapsigargin for 24 h to produce ER stress, (**C**) wild-type cells showed a significant reduction in *Ern1* mRNA rate, but no significant decline in *Atf6* or *Eif2ak3* mRNA levels. (**D**) TXNDC5 knockout cells revealed that the presence of squalene reduced *Eif2ak3* expression when exposed to a compound that generates ER stress, but there was no difference in *Atf6* and *Ern1* expression. The two-way ANOVA and Mann–Whitney’s U-test were used in the data analysis; * *p* < 0.05, ** *p* < 0.01.

To understand the effect of squalene on ER stress markers, we considered whether *Atf6*, *Ern1* and *Eif2ak3* expressions were caused by the squalene presence in mouse hepatocytes. The expression of ER stress-related genes in the presence of 30 µM of squalene loaded in PLGA NPs for 72 h did not show notable differences in AML12 WT cells (Figure 8A). However, in TXNDC5-deficient cells, mRNA abundance of *Atf6* and *Ern1* was significantly up-regulated by squalene treatment; furthermore, there was a significant difference in *Eif2ak3* expression (Figure 8B). When exposed to 12.5 nM of thapsigargin for 24 h to induce ER stress, for evaluating the function of squalene-PLGA NPs, wild-type cells exhibited a substantial attenuation in *Ern1* transcription rate; whereas, *Atf6* and *Eif2ak3* presented no significant decrement in mRNA levels (Figure 8C). The results in TXNDC5 knockout cells indicated that the *Eif2ak3* expression had a profound reduction in the presence of squalene in exposure to agents that cause ER stress, while no difference in *Atf6* and *Ern1* expression was observed between the squalene loaded in PLGA NPs and the PLGA nanoparticles (Figure 8D). These findings suggest that squalene plays an important role in maintaining the viability of mouse hepatocytes by repressing *Ern1* and *Eif2ak3* signaling in wild-type and knockout cells, respectively.

## 4. Discussion

The goals of this study were to vindicate the influence of squalene, the main ingredient of the unsaponifiable fraction in virgin olive oil, loaded into PLGA nanoparticles on oxidative and ER stress in a mouse hepatic cell line. Furthermore, the dependence or independence on TXNDC5 putative molecular pathways involved in hepatic stress has been investigated. In this context, PLGA, as a naturally derived and biodegradable polymer, is being explored for its ability to transport compounds of therapeutic relevance. Squalene is a naturally occurring cholesterol precursor that forms stable colloidal phases in water [54], and itself is a carrier of many substances and, considering the biological action of squalene, the effect could be a sum of actions [55]. As a result, our findings revealed that squalene was effectively encapsulated in PLGA NPs, resulting in stable NPs. Overall, the synthesis of squalene/PLGA NPs using the single-emulsion solvent evaporation method is a simple preparation procedure, which could facilitate their successful clinical translation.

Based on microscopic observations of PLGA NPs encapsulating fluorescent probes and/or the measurement of the probe’s intracellular levels, many investigations have indicated quick and efficient cellular uptake of PLGA NPs [56,57,58]. The assessment of squalene content in our study revealed that PLGA nanoparticles have a great ability to transfer squalene as a nanocarrier in mouse hepatocytes (Figure 3). As described in different studies, PLGA nanoparticles are effective nanocarriers for the encapsulation and delivery of various anti-cancer agents such as oleanolic (OA) and ursolic (UA) acids in three different cell lines, i.e., HepG2 (human hepatoma cell line), Caco-2 (human epithelial colorectal adenocarcinoma cell line) and Y-79 (human retinoblastoma cell line) [16]; also, for the encapsulation of several pharmaceuticals including haloperidol, estradiol, etc. [20]. For instance, the PLGA-based curcumin NPs have displayed entrapment efficiency in the range of 77 to 85% [59].

**Figure 9:**
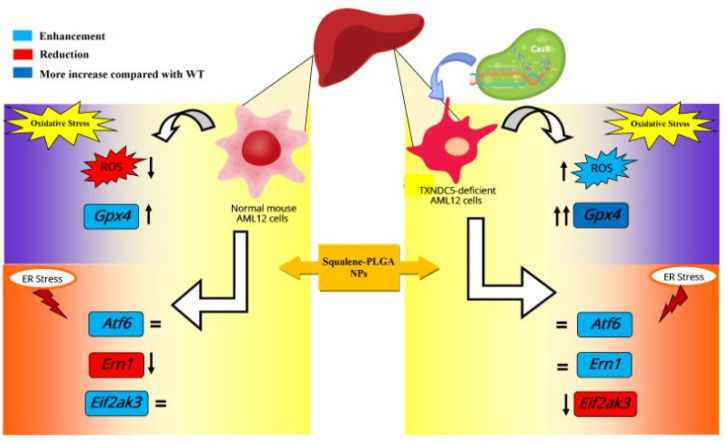
Scheme showing the effects of squalene on cells involved in oxidative and ER stress in the presence and absence of TXNDC5. In presence of squalene, wild-type and TXNDC5 deficient AML12 cell lines were exposed to oxidative and ER stress. The scheme reveals that in absence of TXNDC5, ROS abundance and *Gpx4* mRNA expression are enhanced, and *Eif2ak3* expression is decreased; whereas, in presence of TXNDC5, the ROS amount and *Ern1* mRNA level are reduced. This scheme was designed using Microsoft Publisher Document version 2010. ↑Increased,↓Decreased.

Intracellular ROS production is necessary for regular cellular activities and physiological processes, but when its production exceeds the intrinsic antioxidant capacity, oxidative stress occurs, causing severe damage to cellular macromolecules [60]. Squalene’s antioxidant properties are intimately linked to the unique and stable triterpene structure that allows it to effectively scavenge harmful free radicals [61]. The protective effects of squalene versus oxidative destruction have been formerly documented in rodents [6,62,63,64,65]. Squalene can protect murine macrophages [32], Chinese hamster pulmonary fibroblasts (V79 cells) [66], human monocytes [32] and mammary epithelial cells (MCF10A) from hydrogen peroxide-induced damage in cell culture assays by directly scavenging ROS in a dose-dependent manner [34] and our finding in the present study reveal that squalene can reduce the ROS induction in mouse hepatic cells. Next, we investigated whether TXNDC5 deletion contributes to squalene protection against oxidative stress. ROS influences ER homeostasis and protein folding directly or indirectly, causing ER stress and possibly cell death in the case of extreme ER stress [67,68]. TXNDC5 appears to be involved in the formation of ROS and ER stress, according to growing data [69,70]. Inhibiting TXNDC5 expression via knockdown has previously been shown to induce ROS and ER stress in pancreatic cancer cells [69]; however, increasing TXNDC5 expression in lipid endothelial cells effectively reduces ROS production and protects cells [70]. The present study demonstrates that, in AML12 cells, as reflected in Figure 4A, an inverse and statistically significant difference was found between ROS content and TXNDC5 in exposure to squalene so that, in absence of TXNDC5, ROS was increased, while the ROS was decreased in WT samples. When cells were challenged to an oxidative stimulus (Figure 4B), a significant decrease of ROS in the squalene treated group in WT cells was observed, being the opposite in TXNDC5-KO cells. This finding reinforces previous research that found that oxidative stress-induced TXNDC5 was involved in proper protein folding via its disulfide isomerase activity [71]. Overall, these results evidence that squalene can alter ROS production in oxidative stress, in dependence of TXNDC5 (Figure 9). In the evaluation of the viability of cells in different concentrations of H_2_O_2_ based on the TXNDC5 elimination, in all concentrations that we tested, in the absence of squalene, TXNDC5 deletion significantly reduced the viability of mouse hepatocytes (Figure 5D). However, when the cells were treated with squalene, the viability of both cell lines was increased using 20 and 25 mM concentrations of H_2_O_2_ (Figure 5(D1,D2)). With these results in consideration, TXNDC5 can increase the squalene efficiency in AML12 cell viability and act as an oxidative stress-induced survival factor that regulates ROS/ER stress signaling, allowing AML12 cells to remain viable under oxidative stress, but it is not the only factor, and squalene could bypass it.

Since lipid peroxide accumulation is harmful to cell viability, most mammalian cells repair lipid damage with the phospholipid peroxidase glutathione peroxidase 4 (GPX4), whose blockage results in ferroptosis [72,73,74]. Previous studies have shown that oxidative stress decreased GPX activities and increased MDA accumulation (a substantial product of lipid peroxidation) in plasma and in the liver of piglets [44,75]; whereas our current study results, as shown in Figure 6C,D, confirmed that H_2_O_2_-induced oxidative stress reduced the mRNA level of *Gpx4* in WT and KO mouse hepatic cells. Squalene accumulation in cholesterol auxotrophic lymphomas inhibits oxidative cell death by *Gpx4* induction [76]. Oral administration of squalene improved rats’ redox status after being exposed to cyclophosphamide-induced oxidative stress by boosting GPX activity in the heart, testis and urine bladder and normalize the alteration of *Gpx4* in the heart and hemolysate of red blood cells [62,63]. Our results also evidence the induction of *Gpx4* in the presence of squalene in WT and TXNDC5-deficient cells. When both kinds of cells were exposed to H_2_O_2_, squalene incubation rescued the decreased *Gpx4* expression with independence of TXNDC5 (Figure 6). This mechanism could partly explain that TXNDC5-deficient mouse hepatocyte cells could survive against H_2_O_2_-induced oxidative stress by the therapeutic action of squalene (Figure 9).

TXNDC5 is a thioredoxin with a protein disulfide isomerase-like domain that is expected to catalyze disulfide formation to facilitate protein folding or to control protein function in the face of endoplasmic reticulum stress [77]. When cells were exposed to thapsigargin that compels ER stress, WT and KO cell lines exhibited analogous cell viability (Figure 7A,B). Sullivan et al. found that TXNDC5 could protect endothelial cells from ER stress-induced apoptosis [37]. The major ER stress-induced pro-apoptotic mediators have so far been identified as DDIT3, JNK and cleaved caspase-12. As a downstream protein, DDIT3 is involved in ER stress-induced apoptosis and can be triggered by EIF2AK3, ERN1 and ATF6 [78]. ERN1 is a transmembrane protein that regulates its own expression and functions as a protein kinase and endoribonuclease [79,80]. ATF6, a type II transmembrane protein, is another ER stress sensor, up-regulating chaperones and ERAD pathway components [81], and EIF2AK3 is important for reducing workload by blocking mRNA translation, stopping additional synthesis and, consequently, protein folding under ER stress [82]. To identify the TXNDC5 mediators’ role in endoplasmic reticulum stress, we performed gene expression analysis on *Atf6*, *Ern1* and *Eif2ak3* in the presence of squalene loaded in PLGA (Figure 8). In the context of ER stress-induced effects, Chawsheen et al. found that the knockdown of TXNDC5 in human lung cancer cells accelerates the unfolded proteins and induces ER stress that increases the expression of *Eif2ak3*, *Ern1* and *Atf6* [83].

Accordingly, our results demonstrated that the downregulation of TXNDC5 increased the mRNA level of ER stress markers in mouse hepatic cells (Supplementary Figure S2). In the presence of induced ER stress, squalene decreased *Ern1* expression in WT cells in comparison to KO (Figure 8C). However, the opposite result was observed regarding *Eif2ak3* expression that decreased in TXNDC5 knockout cells, and no significant change was seen in *Atf6* (Figure 8C,D). Multiple experiments showed that knockout of ATF6 prevented the upregulation of *Txndc5* mRNA; TXNDC5 is located downstream of the ATF6 in cardiac, kidney fibroblasts and stelae cells [84,85,86]. Consistent with these results, we found that TXNDC5 may be posited downstream of the ATF6 and upstream of EIF2AK3 and ERN1 in hepatic cells. Based on this hypothesis, we suggest that TXNDC5 may regulate ER activity through distinct signaling pathways in stressful circumstances; moreover, squalene could reduce cell mortality by decreasing *Ern1* or *Eif2ak3* expression as ER stress markers in hepatic cells depending on TXNDC5.

## 5. Conclusions

The current study shows that squalene was successfully encapsulated in PLGA NPs, yielding stable nanoparticles with rapid and efficient cellular uptake. Squalene-based PLGA NPs effectively reduced ROS levels in normal mouse hepatocytes, whereas ROS was increased in TXNDC5-deficient AML12 cells. The cell viability of WT and KO cells under oxidative stress conditions was increased in the presence of squalene by *Gpx4* induction. Squalene also enhanced the cell viability of mouse hepatocytes in thapsigargin-induced ER stress by repressing *Ern1* or *Eif2ak3* expression in wild-type or knockout cells, respectively. Thus, TXNDC5 represented a crucial role in regulating ER activity through different signaling pathways in stressful circumstances. Likewise, squalene-loaded PLGA-NPs protect mouse hepatocytes from oxidative and endoplasmic reticulum stress by variable mechanisms depending on TXNDC5 presence. While this study was successful, there were some limitations in the PLGA encapsulation efficiency of squalene, which was variable in the amount of squalene in each batch of nanoparticle synthesized. Additionally, there was a limitation in PLGA-squalene NP cell treatment, so the cells underwent osmotic stress when exposed to high doses of PLGA-squalene NPs. Hence, high doses of squalene may not be useful in these experiments.

## Figures and Tables

**Table 1:** Squalene content and encapsulation based on the squalene initial volume in PLGA nanoparticles.

Squalene Initial Volume	Squalene Concentration (µM)	Squalene Encapsulation (%)	Squalene/PLGA (*w*/*w*)
100 µL	7472 ± 530	35.8 ± 3.8	0.614
75 µL	5417 ± 474	34.6 ± 3.0	0.445
50 µL	8122 ± 735	77.8 ± 5.1	0.667
25 µL	4842 ± 671	92.7 ± 12.9	0.398

**Table 2:** Physicochemical characterization of PLGA nanoparticles.

Polymer	Diameter (nm)	Dispersion	Zeta Potential (mV)
PLGA NPs with squalene	259 ± 107	0.173	−36.2 ± 0.3
PLGA NPs without squalene	129 ± 37	0.083	−46.8 ± 0.6

## Data Availability

Data is contained within the article and supplementary material.

## Supplementary Materials

The following supporting information can be downloaded at: https://www.mdpi.com/article/10.3390/antiox11030581/s1, Figure S1: Characterization of AML12 cell lines; Figure S2: The mRNA expressions of *Atf6*, *Ern1* and *Eif2ak3* in WT and KO cells; Table S1: Sequences of real-time PCR primers according to MIQE guidelines.

## Abbreviations

PLGA	Poly lactic-co-glycolic acid
NPs	Nanoparticles
ROS	Reactive oxygen species
AML12	Alpha mouse liver cell line
WT	Wild-type
KO	Knock-out
CRISPR	Clustered regularly interspaced short palindromic repeats
MDA-MB-231	M.D. Anderson-metastatic breast 231
ER	Endoplasmic reticulum
UPR	Unfolded protein response
HL-60	Human leukemic cell line 60
PDI	Protein disulfide isomerase
SEM	Scanning electron microscope
TEM	Transmission electron microscopy
DLS	Dynamic light scattering
HPLC	High performance liquid chromatography
MTT	3-(4 5-dimethylthiazol- 2-yl)-2 5-diphenyltetrazolium bromide
DCFH-DA	2,7-dichlorofluorescein diacetate
DCF	Dichlorofluorescein
*Ppib*	Peptidylprolyl isomerase B
*Tbp*	TATA box binding protein
*Gpx4*	Glutathione peroxidase 4
*Eif2ak3*	Eukaryotic translation initiation factor 2 alpha kinase 3
*Atf6*	Activating transcription factor 6
*Ern1*	Endoplasmic reticulum (ER) to nucleus signaling 1
*Txndc5*	Thioredoxin domain containing 5
