# Supplementary material for: Squalene Loaded Nanoparticles Effectively Protect Hepatic AML12 Cell Lines against Oxidative and Endoplasmic Reticulum Stress in a TXNDC5-Dependent Way
Source: Antioxidants (Basel). 2022 Mar 18;11(3):581. doi: 10.3390/antiox11030581 (PMC8945349; doi:10.3390/antiox11030581)
Supplement: Supplementary file 1 [file antioxidants-11-00581-s001.zip › antioxidants-1638961-supplementary.pdf]

Supplementary Table S1. Sequences of real-time PCR primers according to MIQE guidelines

Abbreviations: *Ppib,* peptidylprolyl isomerase B; *Tbp*, TATA box binding protein; *Gpx4*, glutathione peroxidase 4;

Gene symbol	Accession	Primer sequence, sense/antisense ((5'->3')	Amplicon length	Exon	Concentration	Efficiency
Ppib	NM_011149.2	GGAGATGGCACAGGAGGAA<br>TAGTGCTTCAGCTTGAAGTTCTCAT	71	3/4	0.2 µM	99%
Tbp	NM_013684.3	GTGAGTTGCTTGCTCTGTGC<br>GCTGCGTTTTTGTGCAGAGT	359	8	0.2 µM	104%
Gpx4	NM_008162.4<br>NM_001037741.4<br>NM_001367995.1	ATGGATGAAAGTCCAGCCCAAG<br>CGGCAGGTCCTTCTCTATCAC	148	4/7	0.2 µM	96%
Eif2ak3	NM_010121.3<br>XM_011241202.3<br>XM_006505501.2	TATGTTGGAAGGCTTGAGGTCC<br>GTACATTCAGATGCAGCTGTGC	178	13/14	0.2 µM	95%
Atf6	NM_001081304.1<br>XM_030253420.2<br>XM_011238796.4	GGTTCTTCCTCATGGACCAGAT<br>ATAGGTCTGACTCCCAAGGCAT	170	1/3	0.2 µM	102%
Ern1	NM_023913.2	AACAACCTGCCCAAACATCG<br>TGGTCGGTGTGTTGTCTGAA	109	11/12	0.2 µM	93%
Txndc5	NM_001289599.1<br>NM_001289598.1<br>NM_145367.4	CAGGCTTGTCAGATGTCACCAT<br>TAACCTCGTACCGAGTACTTGCTG	82	9/10	0.2 µM	92%

Eif2ak3, eukaryotic translation initiation factor 2 alpha kinase 3; *Atf6*, activating transcription factor 6; *Ern1*, endoplasmic reticulum (ER) to nucleus signaling 1; *Txndc5,* thioredoxin domain containing 5

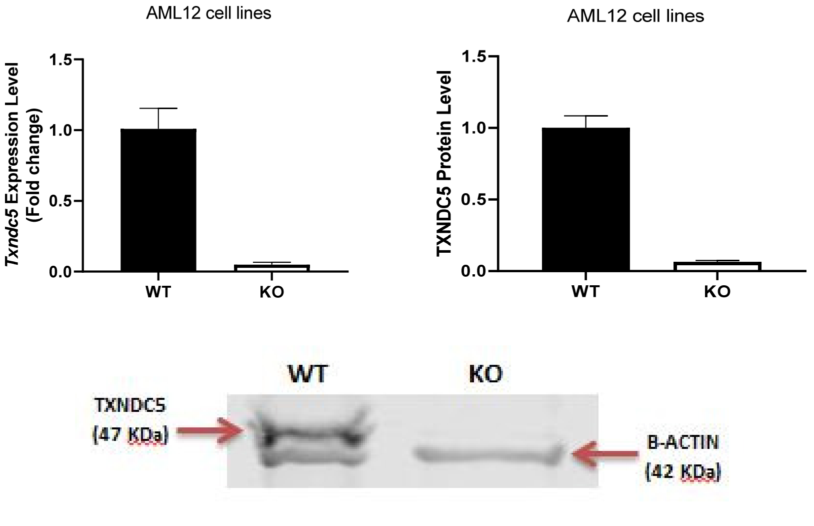

Supplementary Figure S1. Characterization of AML12 cell lines. *Txndc5* mRNA and protein levels in normal mouse hepatocyte AML12 cells (wildtype (WT)) and TXNDC5-deficient AML12 cells (knockout (KO))

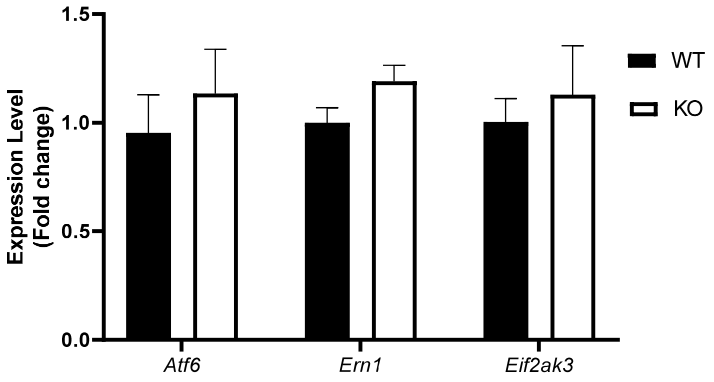

Supplementary Figure S2. The mRNA expressions of *Atf6*, *Ern1*, and *Eif2ak3* in WT and KO cells. Knockout of TXNDC5 increased the mRNA level of ER stress markers.
